# Supplementary material for: Sensitivity of the human temporal voice areas to nonhuman primate vocalizations
Source: eLife. 2026 Sep 3;14:RP108795. doi: 10.7554/eLife.108795 (PMC13541300; doi:10.7554/eLife.108795)
Supplement: Supplementary file 2 [file elife-108795-supp1.docx]

## Supplementary Material

**Table A: Acoustic parameters (N=16) selected among a total of 88 parameters by a General Discriminant Analysis with r<.70 for the assessment of acoustic distance between species.**

| **Acoustic parameter label** | **Acoustic parameter name** |
| --- | --- |
| mfcc4_sma3_amean | Arithmetic mean of the contour of the melodic-frequency cepstral coefficient 4 |
| spectralFlux_sma3_amean***** | Arithmetic mean of the contour of the frequency with most flux around it (change in spectrum) |
| spectralFlux_sma3_stddevNorm | Normalized standard deviation of the contour of the frequency with most flux around it (change in spectrum) |
| equivalentSoundLevel_dBp***** | Conversion of power to decibel of the root mean square of the sound energy |
| slopeV0-500_sma3nz_amean | Arithmetic mean of the slope of timeframe 0-500 |
| mfcc1_sma3_amean | Arithmetic mean of the contour of the melodic-frequency cepstral coefficient 1 |
| F2amplitudeLogRelF0_sma3nz_amean | Arithmetic mean of the second formant amplitude power log relative to fundamental frequency |
| F2amplitudeLogRelF0_sma3nz_stddevNorm | Normalized standard deviation of the contour of the second formant amplitude power log relative to fundamental frequency |
| logRelF0-H1-H2_sma3nz_amean******* | Arithmetic mean of power log relative to fundamental frequency of the harmonic one and two |
| shimmerLocaldB_sma3nz_stddevNorm | Normalized standard deviation of the contour of the relative amplitude difference in local dB |
| loudness_sma3_amean***** | Arithmetic mean of the contour of the sum of the auditory spectrum |
| loudnessPeaksPerSec | Rate of peaks per second of the sum of the auditory spectrum |
| F1amplitudeLogRelF0_sma3nz_stddevNorm | Normalized standard deviation of the contour of the first formant amplitude power log relative to fundamental frequency |
| F2bandwidth_sma3nz_amean****** | Arithmetic mean of the contour of the bandwidth of the second formant |
| shimmerLocaldB_sma3nz_amean******* | Arithmetic mean of the contour of the relative amplitude difference in local dB |
| mfcc2V_sma3nz_stddevNorm | Normalized standard deviation of the contour of the melodic-frequency cepstral coefficient 2 |

**sma3:** smoothed by moving average filter with window length of 3; **sma2nz:** smoothed by moving average filter with window length of 3 for non-zero values. Saturation ([r > .70] and [r < -.70]) of factor: 1*****, 2******, 3*******.

**Table B: Mahalanobis distances estimates, standard errors (SE) and confidence intervals (lower CI: 2.5% and upper CI: 97.5%) for the analyses using generalized linear mixed modelling of factor interaction Species * Social context for acoustic Mahalanobis distances.**

| **Term** | **Estimate** | **SE** | **Lower CI** | **Upper CI** |
| --- | --- | --- | --- | --- |
| *Agonistic ‘threat’* | | | | |
| From human to bonobo | -0.734 | 6.912 | -13.53 | 12.06 |
| From human to chimpanzee | -15.271 | 6.912 | -28.07 | -2.47 |
| From human to macaque | 20.023 | 6.912 | 7.222 | 32.822 |
| *Agonistic ‘distress’* | | | | |
| From human to bonobo | -3.037 | 6.912 | -15.836 | 9.763 |
| From human to chimpanzee | -19.491 | 6.912 | -32.291 | -6.691 |
| From human to macaque | 29.560 | 6.912 | 16.76 | 45.349 |
| *Affiliative ‘positive’* | | | | |
| From human to bonobo | 1.912 | 6.912 | -10.888 | 14.711 |
| From human to chimpanzee | -17.373 | 6.912 | -30.173 | -4.573 |
| From human to macaque | -38.699 | 6.912 | -51.499 | -25.899 |

**Table C:** Activations, cluster size and coordinates for each contrast of interest of model 1 (mean of vocalization fundamental frequency and energy as trial-level covariates of no-interest) in the sample-specific temporal voice areas, wholebrain voxelwise *p*<.05 FDR corrected, k>10.

MNI coordinates

Region label	Hemisphere	X	Y	Z	T value	Cluster size (voxels)

**Chimpanzee > Human, Bonobo, Macaque**

Superior temporal gyrus ant^1^	L	-52	-2	-12	4.84	91

*Superior temporal gyrus mid*	L	-50	-8	-12	4.12

Superior temporal gyrus ant^2^	R	54	0	-12	3.63	18

**Chimpanzee > Bonobo, Macaque**

Superior temporal gyrus ant^3^	R	56	-2	-8	4.89	74

*Superior temporal gyrus mid*	L	-50	-8	-12	4.12

Superior temporal gyrus ant^5^	L	-52	-2	-12	5.18	78

**Chimpanzee > Human**

Superior temporal gyrus ant^4^	L	-50	-4	-12	3.36	74

**Human > Chimpanzee**

Supramarginal gyrus	R	56	-42	28	8.41	5941

*Superior temporal gyrus mid	R	56	-10	2	8.09*

*Superior temporal gyrus post	R	58	-46	14	6.76*

*Superior temporal sulcus post	R	66	-32	0	6.50*

*Middle temporal gyrus mid	R	68	-20	-10	5.52*

*Superior temporal sulcus ant	R	66	-14	-6	5.18*

*Middle temporal gyrus ant	R	60	4	-20	4.45*

Supramarginal gyrus	L	-56	-40	34	7.51	6109

*Superior temporal gyrus mid	L	-50	-18	4	7.37*

*Superior temporal gyrus post	L	-56	-52	18	6.53*

*Middle temporal gyrus mid	L	-64	-22	-10	6.10*

*Middle temporal gyrus post	L	-62	-44	-6	4.87*

*Superior temporal sulcus mid	L	-54	-32	-4	4.76*

ant: anterior; mid: central part; post: posterior.

^1-5^Figure S1 cluster labels

**Table D:** Activations, cluster size and coordinates for each contrast of interest of model 2 (inter-species vocalization acoustic distance as trial-level covariate of no-interest) in the sample-specific temporal voice areas, wholebrain voxelwise *p*<.05 FDR corrected, k>10.

MNI coordinates

Region label	Hemisphere	X	Y	Z	T value	Cluster size (voxels)

**Chimpanzee > Human, Bonobo, Macaque**

Superior temporal gyrus ant^6^	L	-52	-2	-12	4.75	74

**Chimpanzee > Bonobo, Macaque**

Superior temporal gyrus ant^7^	L	-54	-2	-12	5.15	31

Superior temporal gyrus ant^9^	R	56	-2	-8	4.68	30

**Chimpanzee > Human**

Inferior temporal gyrus ant	L	-36	-2	-36	5.54	1310

*Hippocampus	L	-40	-24	-18	4.50*

*Superior temporal gyrus ant^8^	L	-50	-4	-12	3.30	49*

**Human > Chimpanzee**

Supramarginal gyrus	R	56	-42	28	8.91	6411

*Superior temporal gyrus mid	R	56	-8	2	8.62*

*Superior temporal gyrus post	R	58	-44	20	7.80*

*Middle temporal gyrus mid	R	68	-20	-10	5.76*

*Superior temporal sulcus ant	R	58	4	-20	5.00*

Supramarginal gyrus	L	-62	-48	24	7.66	6527

*Superior temporal gyrus mid	L	-50	-18	6	7.50*

*Middle temporal gyrus mid	L	-58	-32	-6	5.24*

*Superior temporal gyrus ant	L	-54	0	2	5.18*

ant: anterior; mid: central part; post: posterior.

^6-9^Figure S2 cluster labels

**Table E:** Acoustic differences between chimpanzee and bonobo calls, per Context, in a dedicated analysis using eGeMaps parameters for these two species.

| **PC** | **Eigenvalue** | **Variance Explained (%)** | **Cumulative Variance (%)** | **GeMAPS Feature (raw)** | **Short Name** | **Dominant Loading** |
| --- | --- | --- | --- | --- | --- | --- |
| PC1 | 2,992 | 12.47 | 12.47 | F0semitoneFrom27.5Hz_sma3nz_pctlrange0.2 | F0 Range (semitones) | +0.349 |
| PC2 | 2,281 | 9.50 | 21.97 | slopeV500.1500_sma3nz_stddevNorm | Spectral Slope 500–1500 Hz Variability | +0.441 |
| PC3 | 2,129 | 8.87 | 30.84 | F1frequency_sma3nz_stddevNorm | F1 Frequency Variability | +0.357 |
| PC4 | 1,771 | 7.38 | 38.22 | StddevUnvoicedSegmentLength | Unvoiced Segment Duration SD | +0.432 |
| PC5 | 1,639 | 6.83 | 45.05 | HNRdBACF_sma3nz_stddevNorm | HNR Variability | -0.339 |
| PC6 | 1,459 | 6.08 | 51.13 | slopeV0.500_sma3nz_amean | Spectral Slope 0–500 Hz (mean) | +0.474 |
| PC7 | 1,394 | 5.81 | 56.94 | F1bandwidth_sma3nz_stddevNorm | F1 Bandwidth Variability | +0.414 |
| PC8 | 1,253 | 5.22 | 62.16 | F0semitoneFrom27.5Hz_sma3nz_stddevRisingSlope | F0 Rising Slope Variability | -0.403 |
| PC9 | 1,22 | 5.09 | 67.24 | F2amplitudeLogRelF0_sma3nz_stddevNorm | F2 Amplitude Variability (rel. F0) | +0.387 |
| PC10 | 1,051 | 4.38 | 71.62 | slopeV0.500_sma3nz_stddevNorm | Spectral Slope 0–500 Hz Variability | +0.358 |
| PC11 | 0,984 | 4.10 | 75.72 | F3amplitudeLogRelF0_sma3nz_stddevNorm | F3 Amplitude Variability (rel. F0) | +0.520 |
| PC12 | 0,906 | 3.77 | 79.50 | jitterLocal_sma3nz_stddevNorm | Jitter Variability | +0.407 |
| PC13 | 0,88 | 3.67 | 83.16 | logRelF0.H1.A3_sma3nz_stddevNorm | H1–A3 Amplitude Ratio Variability | +0.470 |

PC : principal component ; F0 : fundamental frequency ; F1 : first formant ; F2 : second format ; F3 : third formant ; stddev : standard deviation ; Norm : normalized.
