# Supplementary material for: Sensitivity of the human temporal voice areas to nonhuman primate vocalizations
Source: eLife. 2026 Sep 3;14:RP108795. doi: 10.7554/eLife.108795 (PMC13541300; doi:10.7554/eLife.108795)
Supplement: Peer review history

# Editor report: eLife Assessment

**DOI:** 10.7554/eLife.108795.3.sa0

**Authors:** Yanchao Bi

**Affiliations:** Peking University China

**Keywords:** Convincing, Important

This **important** study shows that regions of the human auditory cortex that respond strongly to human voices are also sensitive to vocalizations from closely related primate species. The evidence is **convincing** and methodologically strong. The work offers significant insight into the evolutionary continuity of voice processing and would be of interest to researchers studying auditory processing and evolutionary neuroscience in general.

# Referee report: Reviewer #1 (Public review)

**DOI:** 10.7554/eLife.108795.3.sa1

Summary:

This study investigates how human temporal voice areas (TVA) respond to vocalizations from nonhuman primates. Using functional MRI during a species-categorization task, the authors compare neural responses to calls from humans, chimpanzees, bonobos, and macaques while modeling both acoustic and phylogenetic factors. They find that bilateral anterior TVA regions respond more strongly to chimpanzee than to other nonhuman primate vocalizations, suggesting that these regions are sensitive not only to human voices but also to acoustically and evolutionarily related sounds.

The work provides important comparative evidence for continuity in primate vocal communication and offers a strong empirical foundation for modeling how specific acoustic features drive TVA activity.

Strengths:

(1) Comparative scope: The inclusion of four primate species, including both great apes and monkeys, provides a rare and valuable cross-species perspective on voice processing.

(2) Methodological rigor: Acoustic and phylogenetic distances are carefully quantified and incorporated into the analyses.

(4) Neuroscientific significance: The finding of TVA sensitivity to chimpanzee calls supports the view that human voice-selective regions are evolutionarily tuned to certain acoustic features shared across primates.

(4) Clear presentation: The study is well organized, the stimuli well controlled, and the imaging analyses transparent and replicable.

(5) Theoretical contribution: The results advance u

Comments on revised version.

I thank the authors for having carefully considered and implemented my remarks on the first version.

# Referee report: Reviewer #2 (Public review)

**DOI:** 10.7554/eLife.108795.3.sa2

Summary:

This study investigated how the human brain responds to vocalizations from multiple primate species, including humans, chimpanzees, bonobos, and rhesus macaques. The central finding-that subregions of the temporal voice areas (TVA), particularly in the bilateral anterior superior temporal gyrus, show enhanced responses to chimpanzee vocalizations-suggests a potential neural sensitivity to calls form phylogenetically close nonhuman primates.

Strengths:

The authors employed three analytical models to consistently demonstrate activation in the anterior superior temporal gyrus that is specific to chimpanzee calls. The methodology was logical and robust, and the results supporting these findings appear solid.

Weakness:

The authors only tested vocalizations from three non-human primate species other than humans. In this case, the species specificity of the effect does not fully represent the specificity of evolutionary relatedness.

Comments on revised version.

I have no further comments.

# Referee report: Reviewer #3 (Public review)

**DOI:** 10.7554/eLife.108795.3.sa3

Summary:

Using fMRI, the authors demonstrate that human temporal voice areas (TVA) respond not only to human vocalizations but also to those of other primates, particularly chimpanzee calls, which share acoustic features with human voices. These findings provide compelling evidence for cross-species vocal processing in the human auditory system and carry important theoretical implications for understanding the evolutionary underpinnings of speech perception.

Strengths:

The study offers a valuable comparative design, rigorous acoustic and phylogenetic modeling, and consistent evidence that bilateral anterior TVA regions respond more strongly to chimpanzee vocalizations than to other species' calls. The inclusion of both great apes and monkeys provides a rare cross-species perspective.

Weaknesses:

Minor limitations include the acoustic-phylogenetic confound (which the authors partially address with additional analyses), the lack of non-vocal controls to establish true selectivity.

Overall, the methods, data, and analyses broadly support the claims, with only minor weaknesses that do not undermine the main conclusions. The findings are valuable for the subfield of auditory neuroscience and comparative cognition, with solid evidence supporting the primary claims.

Comments on revised version.

After revision, this work has shown great improvement in data analysis, figure organization, and writing. I have no further suggestions.

# Author comment: Author response

**DOI:** 10.7554/eLife.108795.3.sa4

**Authors:** Leonardo Ceravolo, Coralie Debracque, Thibaud Gruber, Didier Grandjean

**Affiliations:** University of Geneva Geneva Switzerland; University of Geneva Geneva Switzerland; University of Geneva Geneva Switzerland; University of Geneva Geneva Switzerland

The following is the authors’ response to the original reviews.

**Public Reviews:**

**Reviewer #1 (Public review):**

Summary:

This study investigates how human temporal voice areas (TVA) respond to vocalizations from nonhuman primates. Using functional MRI during a species-categorization task, the authors compare neural responses to calls from humans, chimpanzees, bonobos, and macaques while modeling both acoustic and phylogenetic factors. They find that bilateral anterior TVA regions respond more strongly to chimpanzee than to other nonhuman primate vocalizations, suggesting that these regions are sensitive not only to human voices but also to acoustically and evolutionarily related sounds.

The work provides important comparative evidence for continuity in primate vocal communication and offers a strong empirical foundation for modeling how specific acoustic features drive TVA activity.

Strengths:

(1) Comparative scope: The inclusion of four primate species, including both great apes and monkeys, provides a rare and valuable cross-species perspective on voice processing.

(2) Methodological rigor: Acoustic and phylogenetic distances are carefully quantified and incorporated into the analyses.

(4) Neuroscientific significance: The finding of TVA sensitivity to chimpanzee calls supports the view that human voice-selective regions are evolutionarily tuned to certain acoustic features shared across primates.

(4) Clear presentation: The study is well organized, the stimuli well controlled, and the imaging analyses transparent and replicable.

(5) Theoretical contribution: The results advance understanding of the neural bases of voice perception and the evolutionary roots of voice sensitivity in the human brain.

Weaknesses:

(1) Acoustic-phylogenetic confound: The design does not fully disentangle acoustic similarity from phylogenetic proximity, as species co-vary along both dimensions. A promising way to address this would be to include an additional model focusing on the acoustic features that specifically differentiate bonobo from chimpanzee calls, which share equal phylogenetic distance to humans.

(2) Selectivity vs. sensitivity: Without non-vocal control sounds, the study cannot determine whether TVA responses reflect true selectivity for primate vocalizations or general auditory sensitivity.

(3) Task demands: The use of an active categorization task may engage additional cognitive processes beyond auditory perception; a passive listening condition would help clarify the contribution of attention and task performance.

(4) Figures and presentation: Some results are partially redundant; keeping only the most representative model figure in the main text and moving others to the Supplementary Material would improve clarity.

We thank the reviewer for contributing to the improvement of the present study and for the extremely constructive criticism. Concerning the identified weaknesses of our work, we provide here some general answers while the detailed review (below) addresses point-by-point the reviews in high detail.

(1) We totally agree that acoustics and phylogeny cannot be disentangled in our study, which is a limitation. We now provide the suggested analysis on the acoustic specificities of chimpanzee and bonobo calls.

(2) This point on selectivity vs. specificity is indeed crucial, and we now provide a more careful viewpoint and phrasing on this aspect, since our study can only provide partial arguments for this important distinction.

(3) Task demand following species categorization might rightfully yield to the engagement of distinct brain network compared to merely listening to the stimuli. We discuss this aspect and put forward the argument that, while we cannot control for this aspect, our attentional control study performed by an independent sample, N=28 provides clear evidence that no species triggered an attention bias. In other words, task demand might play a role, but at least in the study we know that attentional resources were not biased towards one species in particular since no effects were observed.

(4) We agree that results were not articulated in a clear fashion and that figures were redundant. We addressed this aspect and regrouped the figures where appropriate while we include the rest in the supplementary material now.

**Reviewer #2 (Public review):**

Summary:

This study investigated how the human brain responds to vocalizations from multiple primate species, including humans, chimpanzees, bonobos, and rhesus macaques. The central finding - that subregions of the temporal voice areas (TVA), particularly in the bilateral anterior superior temporal gyrus, show enhanced responses to chimpanzee vocalizations - suggests a potential neural sensitivity to calls from phylogenetically close nonhuman primates.

Strengths:

The authors employed three analytical models to consistently demonstrate activation in the anterior superior temporal gyrus that is specific to chimpanzee calls. The methodology was logical and robust, and the results supporting these findings appear solid.

Weaknesses:

The interpretation of the findings in this paper regarding the evolutionary continuity of voice processing lacks sufficient evidence. A simple explanation is that the observed effects can be attributed to the similarity in low-level acoustic features, rather than effects specific to phylogenetically close species. The authors only tested vocalizations from three non-human primate species, other than humans. In this case, the species specificity of the effect does not fully represent the specificity of evolutionary relatedness.

We want to thank the reviewer for the constructive criticism and for evaluating the manuscript.

Concerning the principal weakness highlighted, we provide new analyses behavioral, acoustics, model-based fMRI that improve our understanding of the influence of both phylogeny and bioacoustics in our data. We argue that the explanation proposed by the reviewer cannot explain our results, as also observed in several other research from us and others. We discuss this aspect and emphasize that including stimuli from more species would greatly improve the understanding of phylogeny and bioacoustics in this context.

**Reviewer #3 (Public review):**

Summary:

Ceravolo et al. employed functional magnetic resonance imaging (fMRI) to examine how the temporal voice areas (TVA) in the human brain respond to vocalizations from different nonhuman primate species. Their findings reveal that the human TVA is not only responsible for human vocalizations but also exhibits sensitivity to the vocalizations of other primates, particularly chimpanzee vocalizations sharing acoustic similarities with human voices, which offers compelling evidence for cross-species vocal processing in the human auditory system. Overall, the study presents intellectually stimulating hypotheses and demonstrates methodological originality. However, the current findings are not yet solid enough to fully support the proposed claims, and the presentation could be enhanced for clarity and impact.

Strengths:

The study presents intellectually stimulating hypotheses and demonstrates methodological originality.

Weaknesses:

(1) The analysis of the fMRI data does not account for the participants' behavioral performance, specifically their reaction times (RTs) during the species categorization task.

(2) The figure organization/presentation requires significant revision to avoid confusion and redundancy.

We thank the reviewer for evaluating our manuscript and for the constructive criticism as well as the many suggestions. Concerning the weaknesses of the study, we provide here some quick answers while more detailed responses can be found below.

(1) We now include behavioral data analysis (accuracy data controlled for reaction times and acoustics of existing Model 3, using mixed-effects logistic regression) in addition to a new, 4th model for fMRI data. This 4th model was computed in a model-based fashion by modeling the probability of correct categorization within the TVA (fitted regression coefficients, per Participant, Species, Trial) and revealing the neural correlates of this modulator.

(2) We totally agree that figure redundancy was a problem and we now reduced confusion by combining congruent aspects while pushing other results to the supplementary material.

**Recommendations for the authors:**

**Reviewing Editor Comments:**

With additional analyses and discussions, the work has the potential to offer important insight into the evolutionary continuity of voice processing.

We thank the Reviewing Editor for this additional motivation and for offering us the possibility to revise our manuscript. We will now provide our point-by-point reviewing, referring to manuscript modifications by section and/or line number(s). All modifications are also highlighted in light grey in the text.

**Reviewer #1 (Recommendations for the authors):**

The manuscript is clearly written and addresses an important comparative question about the specificity of human TVA responses. The acoustic analyses are well designed, and the imaging work is careful and thorough. However, several conceptual and methodological issues need clarification or tempering of claims, particularly regarding (i) the distinction between sensitivity and selectivity, (ii) the confounding of acoustic and phylogenetic factors, and (iii) the interpretation of "chimpanzee-specific" TVA activity.

(1) Introduction

Line 48: cite more recent infant EEG evidence for early voice sensitivity (Calce, Curr Biol).

The reference and explanation were added, lines 46-48.

Line 53: mention recent data on voice processing in marmosets (Jafari, Cell Rep; Dureux, Curr Biol).

We added the references and the mention of these interesting studies on common marmosets, lines 53-54.

Line 59: Fecteau et al. (2004) already explored cross-species selectivity; please integrate and discuss.

We now mention here the work from Fecteau and colleagues and its relevance, see lines 57-59.

Line 70: clarify that in [27] (Bodin et al., 2021) human TVA responded similarly to human nonverbal vocalizations and macaque coos, likely due to acoustic similarity.

We added this important aspect, thank you for this precision. See lines 71-72.

Clarify why an active species-categorization task was chosen instead of passive listening, which is standard in TVA research. Were participants familiarized with stimuli beforehand?

We added a sentence on this aspect, but basically to summarize it here: we wanted to be able to test human recognition of nonhuman primate species’ calls. From the start, we wanted to test the frontal mechanisms related to decision-based processes of humans when categorizing non-human primate calls hence the 2023 article we published. See lines 75-77 and we also added information on familiarization to the stimuli in the Methods, lines 679-682.

The 16 acoustic features mentioned should be briefly defined earlier, as they are central.

We feel like describing 16 acoustic parameters in the introduction would be heavy on the reader, so we instead added a reference to the supplementary table (Table S1) in which these are named and described. See line 80.

Explain why only chimpanzees and bonobos were selected among the great apes, and discuss the value of including both, given their equal phylogenetic proximity but largely dissimilar acoustics.

The stimuli were obtained by Thibaud Gruber and his team and through collaborations with Katie Slocombe and Zanna Clay. Unfortunately, at the time we could only use chimpanzee and bonobo calls for the great apes. Therefore, it was mainly a material constraint rather than a deliberate choice to exclude other great apes. We now discuss this aspect and present the absence of other great apes as a limitation (lines 587-591).

Rephrase references to "recruitment" of TVA - this term implies general activation, while the key question concerns selectivity (stronger responses to voices vs. non-vocal controls).

We rephrased throughout the manuscript, thank you for this suggestion.

The hypothesis section should more clearly separate the acoustic and phylogenetic predictions, and clarify which earlier data motivate each.

We now explicitly categorize the hypotheses according to either Bioacoustics or Phylogeny to clarify. We also added references motivating each hypothesis. See lines 114-120.

(2) Methods

Clarify whether stimuli were RMS-normalized or otherwise balanced for energy (line 128).

Sound pressure level was kept constant but the stimuli were not normalized, specifically to avoid a negative impact on their naturality. We added a sentence (lines 131-132) including a reference on this aspect.

The task design could benefit from reporting accuracy in addition to reaction times for the 4AFC species classification task.

We agree this aspect was missing. We now report accuracy data (controlled for reaction times and acoustics of Model 3) for the species categorization task (lines 147-165; Fig.1B), and in the Methods (lines 769-786). The fitted regression values of this analysis are also used for a new fMRI model (Model 4), to uncover within-TVA correlates of the probability of correct species categorization (lines 309-325; Fig.4).

Please note that previously, the behavioral data of the species categorization task were completely absent (N=23), and the reaction times data previously part of Fig.1 were for the species attentional bias task (independent sample of N=28). Since this aspect was not clear at all (same remark by all reviewers—apologies for that), we now include a clear separation in Fig.1, with newly added panels D & E part of a distinct figure area named: “Control task: Testing for Species attentional bias (N=28)”. Panel D illustrates the control task paradigm (each species as exogenous cue; “dot-probe” paradigm) while panel E shows the results (target sine wave tone or “bip” detection reaction times), showing that no species triggered more attentional capture than the others (Species effect non-significant).

The acoustic parameters used in Models 2 and 3 should be explicitly listed in the Methods (even if already published elsewhere).

In addition to their description in Table S1, we now include the 16 acoustic parameters used to calculate acoustic distance between the species in the Methods, see lines 828-844.

Consider simplifying the presentation of the three models: a figure summarizing their relationships would help.

We now include only one figure (Fig.2) for Model 3, and we pushed model 1&2 to the supplementary material. We also simplified Fig.3 for a clearer view of the overlaps between the 3 models within the TVA.

The description of “systematic and thorough control of phylogeny” (line 119) is overstated, given that only three nonhuman species were included.

We agree with the reviewer and we suppressed both “systematic” and “thorough” from the sentence.

Provide rationale for not including a nonvocal control category (e.g., scrambled vocalizations or environmental sounds) to assess TVA selectivity.

The main objective of the study was to uncover whether human participants could recognize the vocalizations from nonhuman primates—from both great apes and monkeys—as compared to the human voice. We therefore did not include nonvocal or noise stimuli. We added this point as a limitation in the Discussion (lines 593-596 and 609-611).

Even though we did not include such stimuli for the reason mentioned above, the delineation of subtypes of nonvocal material within the TVA of our participants (Fig.2) are, in our opinion, clarifying the message: chimpanzee-selective activations are fully within ‘voice vs. animal’ and ‘voice vs. nature’ TVA subareas, while it is not the case in ‘voice vs. music’ and ‘voice vs. noise’ TVA subareas.

Clarify if participants were trained or had a practice session to recognize the four species before scanning.

The participants were indeed trained on 3 stimuli per species before entering the MRI scanner. These stimuli were discarded from the species categorization task. We added a sentence about this aspect, see lines 131-132.

Specify what is meant by "no good or bad response" in the attentional control task (line 724).

We suppressed this wording as it was highly confusing.

(3) Results

Behavioral accuracy should be reported to complement reaction times.

We now added behavioral data for the species categorization task as well as the neural correlates of accurate species categorization. See our previous response above (‘‘‘).

Figures 2-4 largely overlap; consider merging or simplifying to reduce redundancy.

We agree and this point was raised by the other reviewers as well. Task-based results are now presented only for Model 3 as Fig.2, while Fig.3 (previously Fig.5) summarizes the overlap between the three models. Figures for Models 1 & 2, previously labelled Fig.3 and Fig.4, were moved to the supplementary material.

Figure 2: Please indicate more clearly where "chimp-selective" areas are located (perhaps with zooms).

We agree, we now modified Fig.2 with zoomed-in panels and a clearer outline of chimp-selective areas (solid blue outline). This outline is also referenced in the text (lines 236-237).

Correction for multiple contrasts: With many pairwise tests, adjustments (Bonferroni or FDR) should be mentioned explicitly.

We now specify ‘FDR correction at the voxel level’ at the beginning of the Results section (lines 195-198) as well as in each figure.

Replace "specific to chimpanzee" with "selective for chimpanzee" to avoid implying exclusivity.

We made the suggested replacement throughout the manuscript.

Discuss whether the small macaque-related clusters might simply reflect acoustic overlap rather than true category selectivity.

We added a section on this important aspect, including results that support the role of mid-STG/STS regions for more noise-like stimuli, including the use of macaque coos. See lines 450-461.

(4) Discussion

The discussion overstates claims of "chimpanzee-selectivity" in TVA. The evidence shows relative preference, not absolute selectivity.

We now specify from the start of the Discussion that we are not interpreting the results as absolute selectivity but rather as more relative preference, see lines 371-373.

The authors repeatedly conflate acoustic and phylogenetic factors; this should be explicitly acknowledged as a limitation.

We agree, and we completed the limitations section already dedicated to this aspect by a more explicit account of the confound, see lines 609-611.

Clarify what is meant by "recruitment" and "selectivity" (lines 411-419, 577). TVA activity often reflects enhanced responses to voices compared to non-vocal sounds, not exclusive activation.

We clarified this wording in the Discussion (lines 377-378) and replaced another instance by “activated the […]” to make it clearer what we imply, namely enhanced activity triggered by chimpanzee calls within human TVA.

The lack of non-vocal control conditions should be discussed as a major interpretive limitation.

We added this point as a limitation in the Discussion (lines 593-596).

The statement that "chimpanzee-selective activity" arose in humans who have never been exposed to chimp calls (line 450) invites evolutionary speculation but should be more cautiously phrased.

We agree, and we rephrased by: “[…] with chimpanzee calls triggering responses in the anterior STG/TVA of our human participants […]”. See lines 432-433.

The comparison to recent macaque data (Giamundo et al., 2024 PNAS) is crucial: these findings of human-voice-selective neurons in macaques directly parallel the present human-chimp result.

We agree with the reviewer, and we are hopeful to read similar results for other apes/great apes in the future.

**Reviewer #2 (Recommendations for the authors):**

(1) The primate vocalizations used in this study were recorded in diverse social and emotional contexts, which may have contributed to the observed differences in TVA activation. Since the temporal voice areas are known to be sensitive to affective and socially relevant cues, these contextual differences could confound the interpretation of species-specific neural responses. Therefore, I suggest that the authors conduct a post-hoc analysis to quantify and compare the affective valence, arousal levels, and social contexts associated with each stimulus set.

We agree that the TVA are sensitive to social—or socially relevant—cues, motivating the very thorough work of the expert reserve personnel on-site to accurately categorize the calls according to the very specific context they were produced in. If the reviewer meant presenting these stimuli to non-expert participants and asking them to categorize the context or valence, we think it would make no sense since the ratings would be completely below chance level and therefore uninformative. The newly added behavior—and model-based fmri—data include this crucial point, a factor that we named ‘Context’ in our analyses. In fact, for each species’ 18 stimuli, we control for agonistic and affiliative production context—split evenly, per species. Also, computing an additional posthoc analysis by splitting the stimuli according to Context would result in too few trials to get sensible and reliable fMRI results.

That being said, our study targets this specific aspect by extracting the acoustic features that characterize our stimulus set the best, across context-species-valence-arousal, which is exactly what we want. Through the three types of modeling we used—from more simplistic to more elaborate the results converge only for one species: chimpanzee calls.

We think the addition of behavioral data, model-based fMRI data, and the specific analysis on acoustic differences between chimpanzee and bonobo calls strengthens the message and the validity of our findings.

(2) Although the author mentioned that the behavioral effects triggered by these vocalizations have been reported previously, the behavioral responses of the participants in the current study are also crucial for our understanding of the results. If the MRI data can be combined with the participants' behavioral responses for comprehensive analysis, the conclusions of this study will be more compelling.

We agree with the reviewer, and we added the behavioral data—controlling for reaction times, production context and acoustics of interest—and we also included a model-based fMRI modeling of the probability of correct species categorization as Model 4, Fig.4. See, respectively: lines 147-165, Fig.1B; Methods, lines 769-786; Neuroimaging results, lines 309-325.

(3) I am still not convinced that phylogenetic proximity drives the observed neural selectivity. While chimpanzee vocalizations do elicit stronger responses in anterior STG, the claim that this reflects evolutionary relatedness lacks evidence. If the acoustic features of a certain call from a particular species are similar to those of human voices, it may also lead to similar effects.

We agree with the reviewer that generalizing our results in terms of phylogenetic proximity alone is not a viable option. Including many more primate species including other great apes would be necessary, and we mention this crucial aspect in the limitations section. We also insist in the Discussion on the interdependence between phylogeny and acoustics in our data, since: (1) we cannot fully disentangle these factors here, (2) we cannot attribute our results to either one or the other. See lines 387-390, 410-411, 473-477, 587-591.

If the acoustic features of a certain call from a particular species are similar to those of human voices, it may also lead to similar effects.

We agree, and nobody could disagree: if an auditory object is extremely similar to the human voice in terms of acoustics, it would therefore potentially activate the TVA. This is exactly our message: in the natural ‘auditory world’, the calls from chimpanzees seem to be among the very few animal auditory signals that are sufficiently close, acoustically, to the human voice and therefore trigger TVA activity. They also happen to be the calls from a species which is phylogenetically the closest to humans with minimal differences with other great apes. Our results are in that sense very aligned with work from the laboratory of Pascal Belin, namely on ‘voice patches’ in the primate brain located in the (anterior) TVA, cited in our manuscript.

We therefore think our interpretation does not exclude that in the near future, similar results within the TVA could be observed for other auditory objects, and if animal, from a species potentially much more distant phylogenetically or from vocal signals of other great apes.

We added a key limitation point in the Discussion on the absence of auditory control stimuli in our design, such as scrambled or spectrum shifted per-species stimuli, which would have made the interpretation clearer identical acoustics but alteration/destruction of the species auditory object. See lines 593-596 and 609-611.

**Reviewer #3 (Recommendations for the authors):**

While the manuscript presents intriguing results, several concerns are raised for further consideration, detailed below.

We thank the reviewer for evaluating the manuscript and for the constructive criticism and suggestions.

Major concerns:

(1) This study claims that bilateral anterior superior temporal gyrus (aSTG) in humans can be specifically activated by chimpanzee vocalizations rather than all other primate species after regressing out relevant acoustic parameters using three distinct analyses. I am wondering if a control stimulus (e.g., scrambled chimpanzee vocalizations) were presented, would the activation patterns in these same temporal voice areas (TVA) exhibit significant differences compared to the natural chimpanzee vocalizations?

We completely agree with the reviewer, and this point was also raised by the other reviewers. We therefore added a key limitation point in the Discussion on the absence of auditory control stimuli in our design, such as per-species scrambled or spectrum shifted stimuli, which would have made the interpretation clearer—identical acoustics but alteration/destruction of the species auditory object. See lines 609-611.

(2) The figure organization/presentation requires significant revision to avoid confusion and redundancy. E.g:

Figure 1C is the same as Figure S1. In addition, Figure 1C lacks a figure legend and descriptive label.

The scatter plots in Figures 2D, 2H, 3D, 3H, and 4D, 4H are same as those in Figures S2, S3, and S4. However, some of these duplicate plots even have inconsistent axis labels.

In several panels, the main figures appear to be summaries derived from the supplementary figures. The authors should organize these figures well to eliminate redundancy.

Please double-check all the figures to make sure of accuracy.

We agree that the figures were badly organized and were too crowded and redundant. We now suppressed the redundancy between Fig.1 and Fig.S1, and we reduced fMRI results to one figure for statistical Model 3 while the other models are in the supplementary data—we also justify this decision in the text by highlighting that model 3 is the most elaborate and sensitive one. Fig.3 (previously ‘Fig.5’) shows the overlaps between models and was simplified and clarified as well.

(3) The analysis of the fMRI data does not account for the participants' behavioral performance, specifically their reaction times (RTs) during the species categorization task. It is possible that processing vocalizations from certain species requires more cognitive effort or induces higher decision uncertainty. Could the observed neural effects be confounded by the decision-making process itself?

We now include behavioral data analysis (accuracy data controlled for reaction times and acoustics of existing Model 3, using mixed-effects logistic regression) in addition to a new, 4th model for fMRI data. This 4th model was computed in a model-based fashion by modeling the probability of correct categorization within the TVA (fitted regression coefficients, per Participant, Species, Trial) and revealing the neural correlates of this modulator. We now display these results in Fig.4 and we introduce the motivation factor for including a categorization task rather than more traditional passive listening (lines 75-77), as well as limitations, lines 595-596.

(4) One interesting attempt of this study is to dissociate biologically salient information in animal vocalizations from their low-level acoustic properties. This presents a fundamental conceptual challenge: how to rigorously disentangle a vocalization's species-specific attributes from its inherent acoustic correlates. More precisely, what essential biological information persists in a species' vocal signal after statistically accounting for all quantifiable acoustic features? I recommend that the authors address it in the discussion.

We thank the reviewer for this very important comment, and for suggesting we discuss it in the manuscript. We completely agree: we cannot fully orthogonalize species and acoustics, and this aspect relates also more broadly to cognitive and affective neuroscience studies involving vocal material. Namely: “What is an auditory object without acoustics?”

We included a full paragraph on this aspect, see Discussion, lines 570-584.

(5) If a brain region, such as TVA, is responsive to both acoustic parameters and biological meanings of animal vocalizations, the method used in this study might be inadequate by setting covariates to zero. It is possible that species information is embedded within a specific acoustic pattern. The current modeling approach may not capture such complex information and could potentially introduce bias when estimating the species effect. I recommend that the authors address this issue in the discussion.

We thank the reviewer for this point once again, we addressed it in the Discussion, lines 581-584, and also in the section dedicated to study limitations, lines 609-613.

(6) In the discussion, non-human primate vocalizations are "unreadable" to humans. If this is the case, what is the fundamental perceptual difference between these vocalizations and those from the other animal species? An alternative and highly plausible explanation for the findings is the differential familiarity of the participants with the various species, driven by media exposure (e.g., documentaries) or zoo visits and interactions. The authors need to provide a stronger justification for their control stimuli and directly address, either through discussion or additional analysis, how the factor of familiarity might explain their results better than the proposed "evolutionary distance" hypothesis.

We now discuss this important aspect, see lines 560-569.

We thought about doing additional analyses on this aspect but we concluded that we did not have any reliable indicators of familiarity for our participants, and additionally they were all recruited for being ‘unfamiliar’ with great apes or old-world monkeys’ vocalized communication.

Also, frequent mismatches in the media between images of apes and the associated vocal signals (for instance, the depiction of a chimpanzee but with background audio of macaque coos) are not helping this cause.

Minor:

(1) No figure legend and result description for Figure 1.

Figure 1 has a legend, maybe it was cut out during the uploading process, but it is present and verified now.

(2) In the main text, three statistical models were referenced. Was the data used in each subsequent statistical model derived from the processed data of the preceding model? Please clearly explain this in the main text.

We now specify this aspect in the Methods and the Results section to clarify that each model is independent from the others (lines 964-966 and 189-191, respectively).

(3) In Figure 5, the two dashed lines representing Model 1 and Model 2 are confusing for readers.

We modified the figure (now Fig.3) and simplified it by removing some outlines and clarifying the colors, therefore improving readability.

(4) Lack of reaction times in the species categorization task.

We clarified behavioral data, including the results for the species categorization task and for the control, exogenous cueing task, see modified Fig.1 and behavioral results section of the Results.

(5) Figures 2, 3, 4, 5, Please keep the font size of the figure title consistent.

Figure title font size were uniformized.

(6) Line 201, Line 224, and so on, (EFG) → (E, F, G).

We modified this aspect in every figure legend, including the supplementary material.
